# Supplementary material for: 7,12-Dimethylbenz(a)anthracene as a Model for Ovarian Cancer Induction in Rats
Source: Biology (Basel). 2025 Jan 14;14(1):73. doi: 10.3390/biology14010073 (PMC11762512; doi:10.3390/biology14010073)
Supplement: Supplementary file 1 [file biology-14-00073-s001.zip › biology-3292174-supplementary.pdf]

biology

MDPI

Systematic review and meta-analysis of literature

7,12-dimethylbenz(a)anthracene as a Model for Ovarian Cancer Induction in Rats

Ana Carolina Ciseski Gonçalves [1], Katia Candido Carvalho [2], José Antônio Orellana Turri [2], Ricardo dos Santos Simões [3], Jesus Paula Carvalho [3], Luiz Fernando Ferraz da Silva [4], José Maria Soares Júnior [2,*] and Edmund Chada Baracat [2,3,*]

1 Faculdade de Medicina de Bauru, Universidade de São Paulo, Bauru 17012-901, SP, Brazil; anaciseski@usp.br
2 Laboratório de Ginecologia Estrutural e Molecular (LIM 58) do Departamento de Obstetrícia e Ginecologia, Faculdade de Medicina da Universidade de São Paulo, São Paulo 17012-901, SP, Brazil; carvalhokc@gmail.com (K.C.C.); antonioturri@usp.br (J.A.O.T.).
3 Disciplina de Ginecologia, Departamento de Obstetrícia e Ginecologia, Hospital das Clínicas da Faculdade de Medicina da Universidade de São Paulo, São Paulo 17012-901, SP, Brazil; ricardo.simoes@hc.fm.usp.br (R.d.S.S.); jesuspaulacarvalho@gmail.com (J.P.C.)
4 Departamento de Patologia, Faculdade de Medicina FMUSP, Universidade de São Paulo, São Paulo 17012-901, SP, Brazil; burns@usp.br
* Correspondence: jsoares415@hotmail.com (J.M.S.J.); ecbaracat@gmail.com (E.C.B.); Tel.: +55-11-9820-7578-1 (J.M.S.J.); +55-11-3061-7033 (E.C.B.); Fax: +55-2661-7621 (J.M.S.J.); +55-11-2661-7621 (E.C.B.)

Supplementary Material: Risk of Bias

The Robins 1 tool of PRISMA 2020 was used to assess risk of bias at the studies included in this review. The studies were evaluated by two authors considering the five categories: R: Bias arising from the randomisation process, D: Bias due to deviations from intended interventions, Mi: Bias due to missing outcome data, Me: Bias in measurement of the outcome and S: Bias in selection of the reported result. The summary is presented in the table below (Table S1).

No serious risk of bias was identified in the studies. There were some concerns considering Mi, Me and S. At Cai et al. 2019, Tunca JC. 1985, Seriya S. 1979, Crist KA. 2005; Nishida T. 2000, Hilfrich J. 1975, Jacobs AJ. 1983, Taguchi O. 1988 and Howell JS. 1954 there might be some bias due to missing outcome data (Mi). About Tunca JC. 1985, Craig ZR. 2010, Huang Y. 2012, Nishida T. 2000, Hilfrich J. 1975 and Jacobs AJ. 1983 there were some concerns about bias in measurement of outcomes (Me). Also, Cai et al. 2019, Craig ZR. 2010, Crist KA. 2005, Huang Y. 2012, Nishida T. 2000, Hilfrich J. 1975, Jacobs AJ. 1983 and Howell JS. 1954 seemed to have a moderate bias in selection of reported results (S). Considering the overall bias, though, there were some concerns only about Nishida T. 2000, Hilfrich J. 1975, Jacobs AJ. 1983 as those studies intrigued some questions in three out of five categories of Robins 1.

In consonance with that, the meta analysis of risk of bias showed that there was no high risk of bias. Analyzing all the studies grouped through the Begg's test and a funnel plot, we found out that there is a low risk of bias (less than 1% of bias) for the increased effectiveness and, for mortality, no bias was identified. The Funnel plots are presented below (Figures S1 and S2).

 https://doi.org/10.3390/biology14010073

Table S1. Risk of Bias Summary at Systematic review and meta-analysis of literature DMBA as model for ovarian cancer induction in rat.

Author, year	Country	R: Bias arising from the randomisation process	D: Bias due to deviations from intended interventions	Mi: Bias due to missing outcome data	Me: Bias in measurement of the outcome	S: Bias in selection of the reported result	O: Overall risk of bias.
Cai et al. 2019 [8]	China	+	+	?	+	?	+
Kim KK. 2003 [9]	South Korea	+	+	+	+	+	+
Tunca J C. 1985 [11]	USA	+	+	?	?	+	+
Sekiya S. 1979 [12]	Japan	+	+	?	+	+	+
Yang X Y. 2021 [13]	China	+	+	+	+	+	+
Marion SL. 2013 [14]	USA	+	+	+	+	+	+
Craig ZR. 2010 [15]	USA	+	+	+	?	?	+
Hoyer PB. 2009 [16]	USA	+	?	+	+	+	+
Ting AY. 2007 [17]	USA	+	+	+	+	+	+
Crist KA.	UK	+	+	?	+	?	+

2005 [18]							
Huang Y. 2012 [19]	China	+	+	+	?	?	+
Nishida T. 2000 [20]	Japan	+	+	?	?	?	?
Hilfrich J. 1975 [21]	Germany	+	+	?	?	?	?
Jacobs,A J. 1984 [22]	USA	+	+	?	?	?	?
Taguchi O. 1988 [23]	1988, Japan	+	?	?	+	+	+
Howell J S. 1954 [24]	1954, England	+	+	?	+	?	+

–	+	?
Serious Risk of bias	Low risk of bias	Some concerns

Figure S1: Tests for Publication Bias - Effectiveness

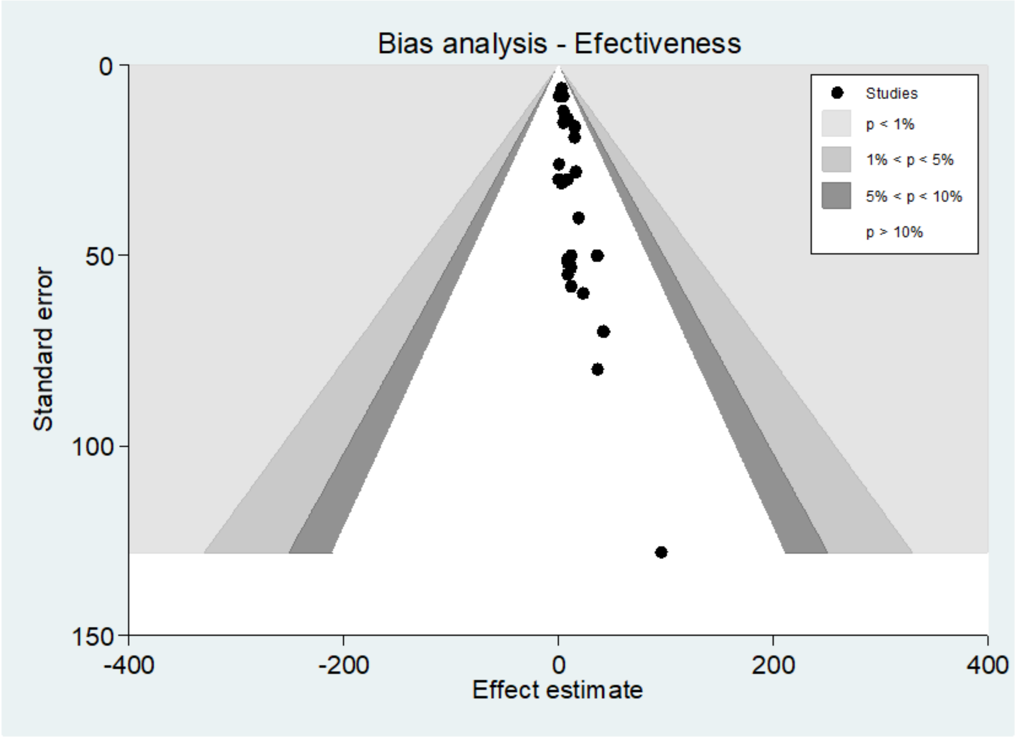

Figure S2: Tests for Publication Bias - Mortality

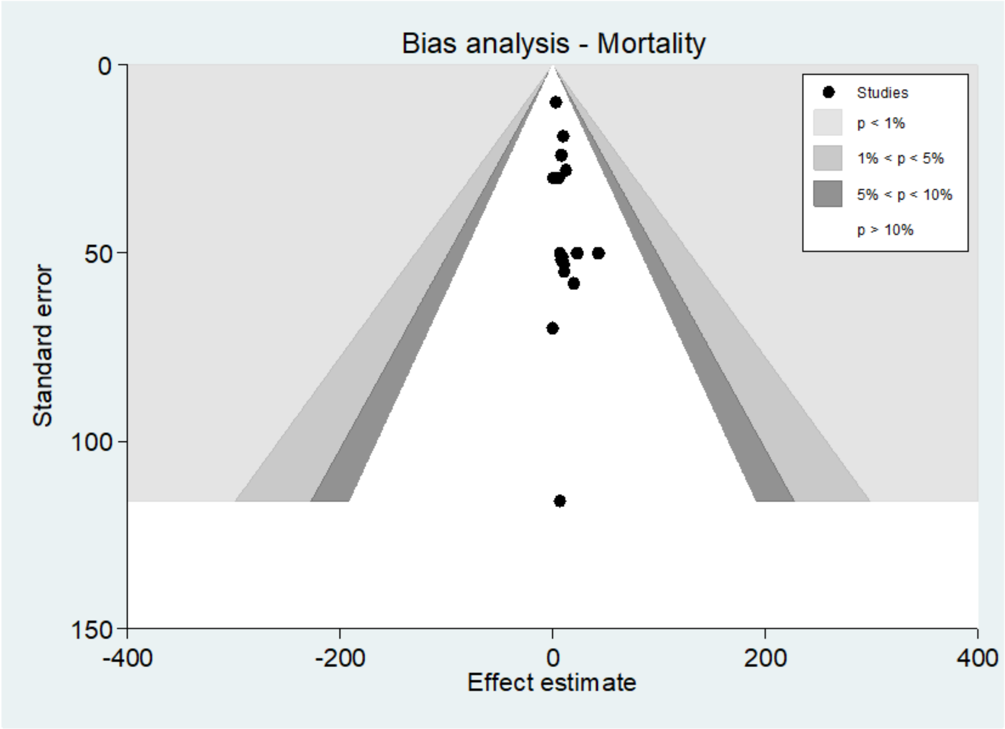

PRISMA 2020 for Abstracts Checklist

Section and Topic	Item #	Checklist item	Reported (Yes/No)
**TITLE**			
Title	1	Identify the report as a systematic review.	Yes
**BACKGROUND**			
Objectives	2	Provide an explicit statement of the main objective(s) or question(s) the review addresses.	Yes
**METHODS**			
Eligibility criteria	3	Specify the inclusion and exclusion criteria for the review.	Yes
Information sources	4	Specify the information sources (e.g. databases, registers) used to identify studies and the date when each was last searched.	Yes
Risk of bias	5	Specify the methods used to assess risk of bias in the included studies.	Yes
Synthesis of results	6	Specify the methods used to present and synthesise results.	Yes
**RESULTS**			
Included studies	7	Give the total number of included studies and participants and summarise relevant characteristics of studies.	Yes
Synthesis of results	8	Present results for main outcomes, preferably indicating the number of included studies and participants for each. If meta-analysis was done, report the summary estimate and confidence/credible interval. If comparing groups, indicate the direction of the effect (i.e. which group is favoured).	Yes
**DISCUSSION**			
Limitations of evidence	9	Provide a brief summary of the limitations of the evidence included in the review (e.g. study risk of bias, inconsistency and imprecision).	Yes
Interpretation	10	Provide a general interpretation of the results and important implications.	Yes
**OTHER**			
Funding	11	Specify the primary source of funding for the review.	Yes
Registration	12	Provide the register name and registration number.	Yes

From: Page MJ, McKenzie JE, Bossuyt PM, Boutron I, Hoffmann TC, Mulrow CD, et al. The PRISMA 2020 statement: an updated guideline for reporting systematic reviews. BMJ 2021;372:n71. doi: 10.1136/bmj.n71. This work is licensed under CC BY 4.0. To view a copy of this license, visit https://creativecommons.org/licenses/by/4.0/

PRISMA 2020 Checklist

Section and Topic	Item #	Checklist item	Location where item is reported
**TITLE**			
Title	1	Identify the report as a systematic review.	Subtitle.
**ABSTRACT**			
Abstract	2	See the PRISMA 2020 for Abstracts checklist.	Supplements.
**INTRODUCTION**			
Rationale	3	Describe the rationale for the review in the context of existing knowledge.	56-72
Objectives	4	Provide an explicit statement of the objective(s) or question(s) the review addresses.	77-79
**METHODS**			
Eligibility criteria	5	Specify the inclusion and exclusion criteria for the review and how studies were grouped for the syntheses.	95-99
Information sources	6	Specify all databases, registers, websites, organisations, reference lists and other sources searched or consulted to identify studies. Specify the date when each source was last searched or consulted.	90-92
Search strategy	7	Present the full search strategies for all databases, registers and websites, including any filters and limits used.	88-90
Selection process	8	Specify the methods used to decide whether a study met the inclusion criteria of the review, including how many reviewers screened each record and each report retrieved, whether they worked independently, and if applicable, details of automation tools used in the process.	99-102
Data collection process	9	Specify the methods used to collect data from reports, including how many reviewers collected data from each report, whether they worked independently, any processes for obtaining or confirming data from study investigators, and if applicable, details of automation tools used in the process.	103-105
Data items	10a	List and define all outcomes for which data were sought. Specify whether all results that were compatible with each outcome domain in each study were sought (e.g. for all measures, time points, analyses), and if not, the methods used to decide which results to collect.	103-105
	10b	List and define all other variables for which data were sought (e.g. participant and intervention characteristics, funding sources). Describe any assumptions made about any missing or unclear information.	103-114
Study risk of bias assessment	11	Specify the methods used to assess risk of bias in the included studies, including details of the tool(s) used, how many reviewers assessed each study and whether they worked independently, and if applicable, details of automation tools used in the process.	115-125
Effect measures	12	Specify for each outcome the effect measure(s) (e.g. risk ratio, mean difference) used in the synthesis or presentation of results.	115-125
Synthesis methods	13a	Describe the processes used to decide which studies were eligible for each synthesis (e.g. tabulating the study intervention characteristics and comparing against the planned groups for each synthesis (item #5)).	94-102
	13b	Describe any methods required to prepare the data for presentation or synthesis, such as handling of missing summary statistics, or data conversions.	No methods required.
	13c	Describe any methods used to tabulate or visually display results of individual studies and syntheses.	No methods required.
	13d	Describe any methods used to synthesize results and provide a rationale for the choice(s). If meta-analysis was performed, describe the model(s), method(s) to identify the presence and extent of statistical heterogeneity, and software package(s) used.	109-130
	13e	Describe any methods used to explore possible causes of heterogeneity among study results (e.g. subgroup analysis, meta-regression).	126-130
	13f	Describe any sensitivity analyses conducted to assess robustness of the synthesized results.	Supplements.
Reporting bias assessment	14	Describe any methods used to assess risk of bias due to missing results in a synthesis (arising from reporting biases).	Not applicable.
Certainty	15	Describe any methods used to assess certainty (or confidence) in the body of evidence for an outcome.	116-120

PRISMA 2020 Checklist

Section and Topic	Item #	Checklist item	Location where item is reported
assessment			
**RESULTS**			
Study selection	16a	Describe the results of the search and selection process, from the number of records identified in the search to the number of studies included in the review, ideally using a flow diagram.	136-138
	16b	Cite studies that might appear to meet the inclusion criteria, but which were excluded, and explain why they were excluded.	Not applicable.
Study characteristics	17	Cite each included study and present its characteristics.	Table 1
Risk of bias in studies	18	Present assessments of risk of bias for each included study.	284-303 + Supplements
Results of individual studies	19	For all outcomes, present, for each study: (a) summary statistics for each group (where appropriate) and (b) an effect estimate and its precision (e.g. confidence/credible interval), ideally using structured tables or plots.	Figures 3-10 and Tables 2-9
Results of syntheses	20a	For each synthesis, briefly summarise the characteristics and risk of bias among contributing studies.	284-303 + Supplements
	20b	Present results of all statistical syntheses conducted. If meta-analysis was done, present for each the summary estimate and its precision (e.g. confidence/credible interval) and measures of statistical heterogeneity. If comparing groups, describe the direction of the effect.	Item 3.1
	20c	Present results of all investigations of possible causes of heterogeneity among study results.	Item 3.1
	20d	Present results of all sensitivity analyses conducted to assess the robustness of the synthesized results.	Item 3.1
Reporting biases	21	Present assessments of risk of bias due to missing results (arising from reporting biases) for each synthesis assessed.	Not applicable.
Certainty of evidence	22	Present assessments of certainty (or confidence) in the body of evidence for each outcome assessed.	Figures 3-10 and Tables 2-9
**DISCUSSION**			
Discussion	23a	Provide a general interpretation of the results in the context of other evidence.	253-268
	23b	Discuss any limitations of the evidence included in the review.	277-282
	23c	Discuss any limitations of the review processes used.	277-282
	23d	Discuss implications of the results for practice, policy, and future research.	351-374
**OTHER INFORMATION**			
Registration and protocol	24a	Provide registration information for the review, including register name and registration number, or state that the review was not registered.	394
	24b	Indicate where the review protocol can be accessed, or state that a protocol was not prepared.	396
	24c	Describe and explain any amendments to information provided at registration or in the protocol.	Not applicable.
Support	25	Describe sources of financial or non-financial support for the review, and the role of the funders or sponsors in the review.	394-401
Competing	26	Declare any competing interests of review authors.	401

PRISMA 2020 Checklist

Section and Topic	Item #	Checklist item	Location where item is reported
interests			
Availability of data, code and other materials	27	Report which of the following are publicly available and where they can be found: template data collection forms; data extracted from included studies; data used for all analyses; analytic code; any other materials used in the review.	All references are cited and have open access.

From: Page MJ, McKenzie JE, Bossuyt PM, Boutron I, Hoffmann TC, Mulrow CD, et al. The PRISMA 2020 statement: an updated guideline for reporting systematic reviews. BMJ 2021;372:n71. doi: 10.1136/bmj.n71. This work is licensed under CC BY 4.0. To view a copy of this license, visit https://creativecommons.org/licenses/by/4.0/
